# CT-based renal and body-composition radiomics model to improve the detection ability of diabetic kidney disease in patients with type 2 diabetes mellitus

**DOI:** 10.3389/fendo.2026.1904758

**Published:** 2026-08-03

**Authors:** Baoli Hao, Honghao Sun, Zimeng Yang, Jinlei Fan, Liping Zuo, Peng Du, Cheng Li, Wangshu Cai, Jiqing Li, Peng Zhou, Guoqiang Tian, Dexin Yu

**Affiliations:** 1 Department of Radiology, Qilu Hospital of Shandong University, Jinan, China; 2 Department of Radiology, Yucheng Hospital of Traditional Chinese Medicine, Dezhou, China; 3 Department of Radiology, Jinan Central Hospital, Jinan, China; 4 Department of Emergency Medicine, Qilu Hospital of Shandong University, Shandong Provincial Clinical Research Center for Emergency and Critical Care Medicine, Jinan, China; 5 Department of Radiology, Shandong Hexin Hospital Co., Ltd., Weifang, China; 6 Qilu Medical Imaging Research Institute, Shandong University, Jinan, China

**Keywords:** body composition, computed tomography, diabetes, diabetic kidney disease, perirenal fat, radiomics

## Abstract

**Objectives:**

Conventional imaging modalities exhibit limited capability in distinguishing diabetic kidney disease (DKD), but radiomics could expand image-derived information. Kidney, perirenal tissue and body composition, whether considered in macroscopic or microscopic profile, are associated with kidney function. The present study seeks to evaluate the efficacy of computed tomography (CT)-derived radiomic features of the kidney and body composition in detecting DKD among individuals with type 2 diabetes mellitus (T2DM).

**Methods:**

Patients with T2DM who underwent abdominal CT and renal function test at two institutions were enrolled. Participants from one institution (n = 256) were randomly allocated into training and internal validation cohorts, whereas individuals from another institution constituted the external validation cohort (n = 64). Two- and three-dimensional radiomics features were retrieved from kidneys, perirenal and renal sinus fat, visceral and subcutaneous fat, and skeletal muscles. Radiomics, clinical, and integrated models were developed and evaluated using the area under the receiver operating characteristic curve (AUC), calibration curves and decision curve analysis.

**Results:**

The radiomics model achieved impressive AUCs of 0.869 (95% CI, 0.809–0.929), 0.831 (95% CI, 0.721–0.940), and 0.833 (95% CI, 0.687–0.979) in the training cohort, internal and external validation cohorts, respectively. The combined model outperformed the other two models, achieving AUCs (95% CI) of 0.913 (0.868–0.957), 0.919 (0.849–0.989), and 0.867 (0.762–0.973) in the training, internal validation, and external validation cohorts, respectively.

**Conclusion:**

CT-based renal and perirenal radiomics features demonstrate strong efficacy in identifying DKD in patients with T2DM.

## 1 Introduction

Diabetic kidney disease (DKD) represents a significant etiology of end-stage renal disease and is linked to excess adverse outcomes in diabetic patients (1, 2). Accumulating evidence highlights the importance of prompt diagnosis and intervention for renal impairment in patients with T2DM to optimize outcomes. Kidney biopsy represents the definitive method for confirming kidney diseases but is limited by its invasiveness and poor reproducibility. Current clinical guidelines recommend that detection of chronic kidney disease primarily relies on urinary or serum biomarkers, despite potential issues with insufficient sensitivity. Renal imaging techniques such as computed tomography (CT) can reveal morphology, but it remains unclear whether any imaging characteristics could reflect the subtle structural and functional alterations of DKD (3).

Recent years have seen the booming of radiomics, which enables the extraction of phenotypic features imperceptible to the unaided eye from standard-of-care images, including shape, texture, intensity, wavelet, and Laplacian of Gaussian filters, thereby providing more comprehensive information than conventional radiological indices (4). In the field of magnetic resonance imaging, Ma et al. innovatively expanded the arterial spin labeling (ASL) examination to obtain radiomics texture attributes in ASL imaging and developed a radiomics model that can predict early renal impairment and progression in patients with T2DM (5). Zha et al. developed a model that incorporates microcirculation-based multi-sequence magnetic resonance imaging radiomics with clinical variables for assessment of kidney fibrosis in chronic kidney disease (6). By capturing complementary information related to renal microcirculation and oxygenation, this approach may hold great potential for individualized assessment of RF and longitudinal monitoring. Prior ultrasound radiomic studies demonstrated that a multi-modality ultrasound model combining shear wave elastography, grayscale ultrasound radiomics, and color Doppler ultrasound radiomics enhances non-invasive renal fibrosis assessment (7). Ultrasound-based radiomics has demonstrated the capability to differentiate varying degrees of renal fibrosis and has exhibited strong diagnostic performance for stages G1 through G3 (8, 9). Ultrasound is safe and widely applicable but exhibits a certain level of subjectivity when acquiring cross-sectional images. CT has the advantage of simultaneously visualizing all tissue structures within the scan field, making it well-suited for monomodal opportunistic, combined multi-organ omics assessments.

Body composition could reflect a series of health conditions. The prognostic utility of CT-based quantitative body composition analysis has been extensively validated across various medical domains, including oncology, chronic obstructive pulmonary disease, and critical care (10, 11). Chronic kidney disease frequently accompanies systemic metabolic and immune disorders that ultimately change muscle mass and function to cause sarcopenia (12). Abdominal adiposity has been demonstrated to have significant associations with DKD, and various abdominal fat depots divided by anatomical locations may confer different metabolic risks and value in DKD assessment (13). Given anatomical proximity, perirenal adipose tissue (PRAT) and renal sinus adipose tissue (RSAT) are considered active contributors to kidney injury. It was reported that PRAT deposition was associated with declined renal function and had a higher predictive value for chronic kidney disease than total, subcutaneous, or visceral fat in T2DM (14, 15). The accumulation of RSAT that wraps the renal arteries and veins is associated with an elevated risk of renal impairment in individuals with overweight, regardless of T2DM (14, 16). These investigations commonly utilize radiodensity, volumetric measurements, or cross-sectional areas at the third lumbar vertebra for quantification and analysis. However, the histopathological manifestations of adipose, muscle tissue, and kidney in diabetes and chronic kidney disease are so complex that straightforward quantification may not adequately capture them. The advancement of radiomics, as mentioned above, has the potential to enhance diagnostic imaging capabilities (4). Nevertheless, to date, no study has reported a fused renal and body composition radiomics model for assessment of DKD risk.

In all, the present study assumes that muscle, fat and renal radiomics features are associated with existing DKD and aims to establish a clinic-radiomics model and assess the value of CT-based radiomics features derived from kidneys and body composition in the recognition of DKD in patients with T2DM. Hopefully, with multi-dimensional data mining, patients with T2DM who undergo abdominal CT for various clinical targets can acquire an opportunistic screen for DKD, thereby adding incremental radiological value for the non-invasive diagnosis of DKD.

## 2 Materials and methods

### 2.1 Participants

**Figure 1:**
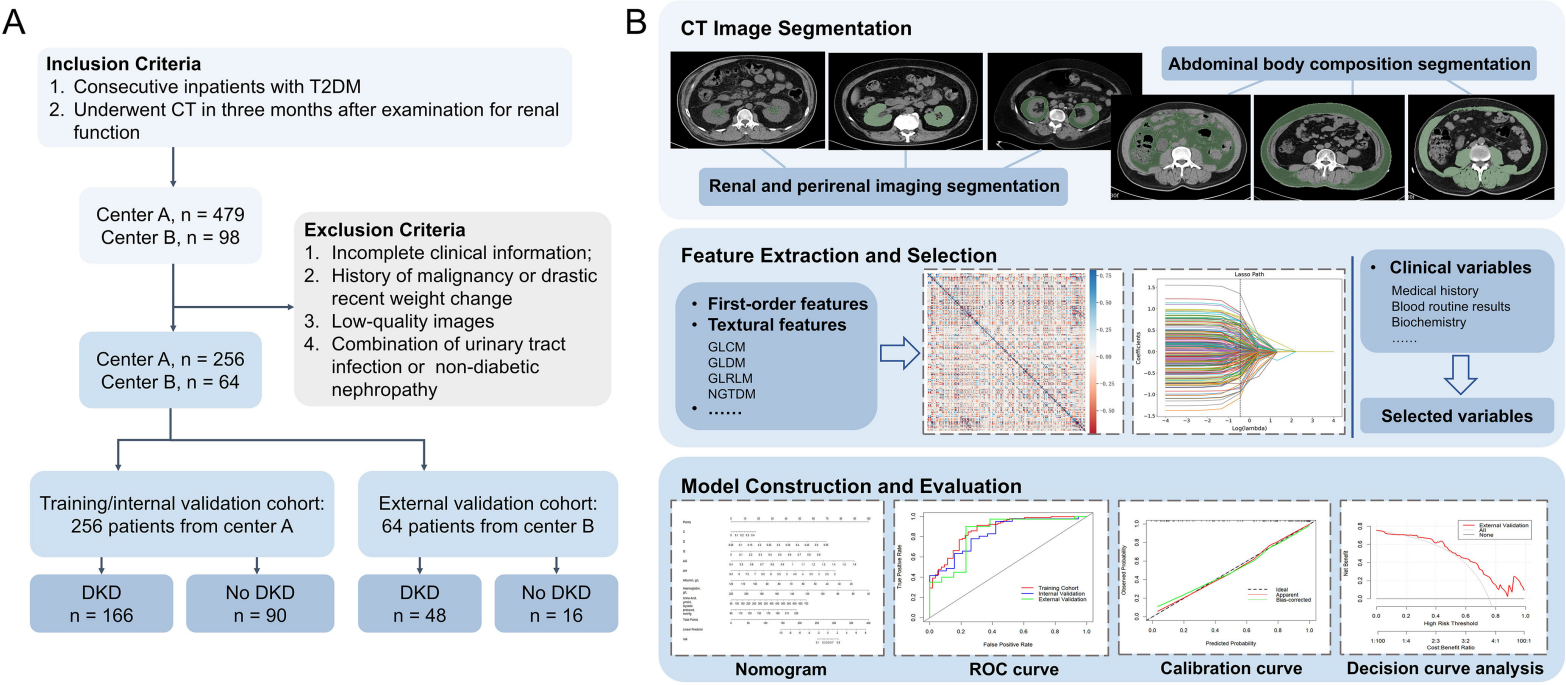
**(A)** Flowchart of the study. **(B)** The workflow of this study. Images were obtained and semi-automatically segmented on 3D slicer. We then utilized the Shukun platform to extract and preselect radiomics features. The resultant features and clinical variables were separately screened to establish radiomics, clinical models and a combined model. Nomograms, ROC curves, calibration curves, and decision curve analyses were performed for model assessment. ROC, receiver operating characteristic; T2DM, type 2 diabetes mellitus; CT, computed tomography; DKD, diabetic kidney disease.

Consecutive inpatients with T2DM with or without DKD who underwent abdominal CT scans within three months after examination for renal function from January 2019 to December 2021 at two medical institutions were included in the study. 479 patients from center A and 98 patients from center B met the above criteria. Given the limited use of renal biopsy in clinical practice, diagnosis of DKD was established if urine albumin-to-creatinine ratio (UACR) ≥ 30 mg/g and/or estimated glomerular filtration rate (eGFR) < 60 ml·min^-^¹·(1.73 m²)^-^¹, presenting for a minimum of 3 months, while excluding nondiabetic causes of kidney diseases (17–19). Afterwards, patients with a) incomplete clinical information, b) history of malignancy, c) drastic recent weight change, d) unsatisfactory image quality, or e) combination of urinary tract infection or non-diabetic nephropathy were excluded from the research. Ultimately, 320 patients were enrolled in model construction and evaluation: 256 from center A were randomly divided into a training cohort and an internal validation cohort at a 3:1 ratio, and 64 from center B constituted the external validation cohort. Clinical and laboratory data were obtained from the hospital information system. Abdominal CT images were acquired from the Picture Archiving and Communication System. The entire participant selection process is shown in **Figure 1A**.

### 2.2 Imaging acquisition and segmentation

**Figure 2:**
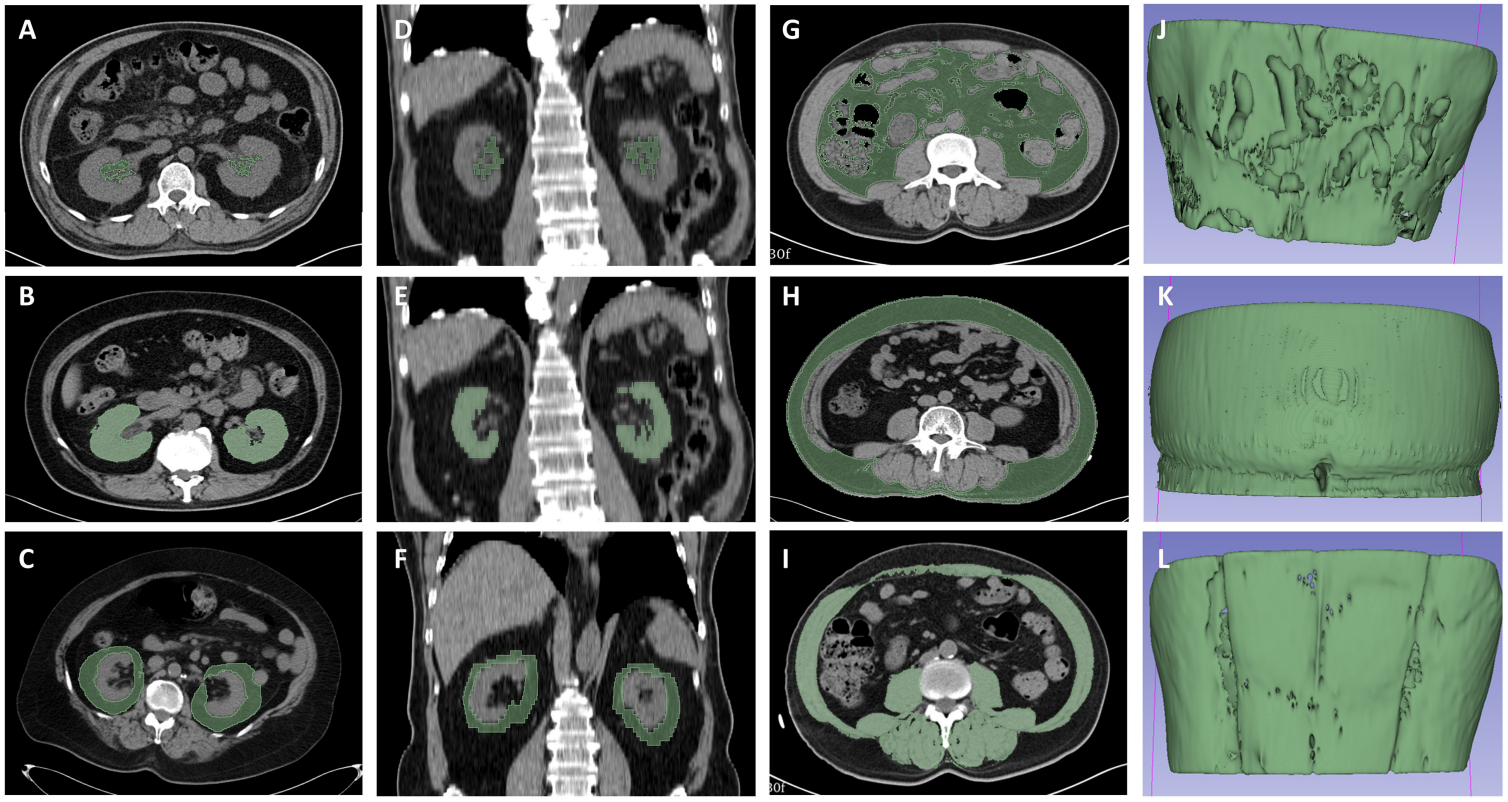
A schematic diagram showing modules of interest. **(A)** renal sinus adipose areas at the level of renal hilum; **(B)** kidney areas at the level of renal hilum; **(C)** perirenal adipose areas at the level of renal hilum; **(D)** renal sinus adipose volumes; **(E)** kidney volumes; **(F)** perirenal adipose volumes; **(G)** visceral fat area at the 3^rd^ lumbar vertebra level; **(H)** subcutaneous fat area at the same level; **(I)** skeletal muscle area at the same level; **(J)** reconstruction view of visceral fat volume between diaphragm and sacrum; **(K)** subcutaneous fat volume; **(L)** skeletal muscle volume.

Abdominal plain CT was performed using a 64-row spiral CT scanner (Siemens Healthcare Definition AS, Germany). The scan parameters were as follows: tube voltage, 120 kV; tube current, 150 mA; pitch, 1 mm; layer pitch, 5 mm; layer thickness, 5 mm; and tube rotation rate, 0.5 s. Abdominal CT scans were completed with the patients lying flat and under breath-holding. Images in Digital Imaging and Communications in Medicine format were semi-automatically delineated by 2 trained radiologists with 8 and 9 years of experience in abdominal radiological diagnosis, with clinical information concealed, using 3D Slicer version 5.6.1. Inter-observer agreement was assessed using the intraclass correlation coefficient (ICC) analysis, and ICC values > 0.8 were considered appropriate for further analysis. In total, regions of interest (ROI) include skeletal muscle (SM), subcutaneous adipose tissue (SAT), and visceral adipose tissue (VAT) at the 3rd lumbar vertebra, and bilateral perirenal adipose tissue (PRAT), renal sinus adipose tissue (RSAT), and the kidney at the level of the renal vein. Additionally, volumes of interest (VOIs) for these anatomical structures were segmented. Specifically, the volumes of SM, SAT, and VAT were delineated from the first to the fifth lumbar vertebrae. PRAT was outlined along the perirenal fascia, while RSAT and the kidneys were segmented within the anatomical range extending from the diaphragm to the first sacral vertebra. Tissues were identified according to both anatomical location and Hounsfield unit (HU). The cut-off values of these segmentations are summarized in **Supplementary Table 1**. A schematic diagram of ROIs and VOIs in this research is shown in **Figure 2**.

### 2.3 Radiomics workflow

The workflow of this research is shown in **Figure 1B**. To ensure uniform voxel spacing and reduce variability, all CT images were resampled to 1×1×1 mm³ isotropic voxels using the linear interpolation algorithm before radiomic feature extraction (20, 21). Features were extracted separately from 6 ROIs and 6 VOIs on each examination using PyRadiomics embedded in the research platform (‘UltraScholar’ Version 2.0, Shukun Technology Co., Ltd, Beijing, China). The extracted features comprised first-order characteristics, including histogram and morphological attributes, as well as second-order parameters. The second-order parameters primarily encompassed metrics derived from the Gray Level Co-Occurrence Matrix (GLCM), Gray Level Dependence Matrix (GLDM), Gray Level Run Length Matrix (GLRLM), Gray Level Size Zone Matrix (GLSZM), and Neighboring Gray Tone Difference Matrix (NGTDM). All the subsequent feature selection processes were performed exclusively on the training cohort to avoid data leakage. The features were initially screened to determine those most pertinent to the presence of DKD. Firstly, features were preselected by excluding those with an absolute Pearson correlation coefficient ≥ 0.95 to minimize redundancy. Subsequently, SelectKBest was applied to identify the *K* features with the highest scores, and then the least absolute contraction and selection operator (LASSO) was used to obtain the 10 most significant features. In the compartment-based screening, Z-score normalization was performed independently for all two-dimensional features and all three-dimensional volumetric features. Original values of the selected features based on each compartment were used when combining these features. In the second round of LASSO reduction, Z-score normalization was performed to these features before LASSO regression. For subunits yielding fewer than ten features through LASSO selection, all identified features were preserved for further analysis. For modules lacking valid variables following LASSO, we selected the ten variables exhibiting the highest absolute K scores, as determined by SelectKBest, to proceed to further screening. These separately selected features were merged and screened again using the LASSO algorithm and 10-fold cross-validation to determine the optimal regularization parameter according to the area under the curve (AUC), followed by univariate and multivariate logistic regression to decide the final modeling features.

### 2.4 Model construction and evaluation

The clinical model was constructed with univariate and multivariate logistic regression analysis. Clinical factors related to the diagnosis of DKD were excluded in variable screening and model construction. Variables demonstrating statistical significance (p < 0.05) in the univariate regression analysis were retained for inclusion in the multivariate model. Subsequently, factors exhibiting significance at p < 0.05 in multivariate logistic regression were selected as the final variables for model construction. Furthermore, a combined model was established by integrating these clinical variables with radiomic features.

The predictive performance of each model was evaluated using the receiver operating characteristic curve (ROC) and AUC for the training, internal validation, and external validation cohorts. DeLong’s test was utilized to statistically compare the predictive performance across models based on their AUC values. Calibration curves were plotted to evaluate the agreement between the model-predicted probabilities and the observed outcomes using the bootstrap resampling method within the training cohort as well as the two validation cohorts. Subsequently, decision curve analysis (DCA) was performed to quantify the net clinical benefits of the radiomics, clinical, and combined models across both the training and validation cohorts, thereby assessing their potential clinical utility. The clinical utility of each model was assessed by calculating its sensitivity, specificity, positive predictive value, negative predictive value, and Youden Index.

### 2.5 Statistical analysis

Data normality was assessed using SPSS version 26.0. Model development and evaluation were carried out utilizing R version 4.3.3. Data normality was evaluated through the Kolmogorov-Smirnov test and histograms. Continuous variables that follow a normal distribution were presented as “mean ± standard deviation”. Continuous variables of skewed distribution were described as “median (Q1, Q3)”. Categorical variables are presented as counts with proportions (%). Comparisons between groups for continuous variables were conducted using the Analysis of Variance, Student’s t-test, Mann–Whitney U test, or Kruskal–Wallis H test, and for categorical variables, using the χ^2^ test. A p-value < 0.05 was considered statistically significant.

## 3 Results

### 3.1 Patient characteristics

The descriptive statistics of the population are shown in **Supplementary Table 1**. Among 320 participants, 106 (33.1%) were not diagnosed with DKD, while 214 (66.9%) were. No statistically significant difference was observed in age (p = 0.972), diastolic pressure (p = 0.301), waist circumference (p = 0.744), LDL-C levels (p = 0.601), HDL-C levels (p = 0.627), BMI (p = 0.173), or smoking status (p = 0.223). Comparatively, patients with DKD had higher systolic pressure (p < 0.001), urine acid (p < 0.001). serum creatinine (p < 0.001), cystatin C (p < 0.001), urinary albumin (p < 0.001), UACR (p < 0.001), and NLR (p < 0.001). This population had lower haemoglobin (p < 0.001) and albumin (p < 0.001) levels in comparison with the no-DKD group. Diabetes duration was longer in those with DKD (p = 0.007). Hypertension (p < 0.001) and a higher proportion of carotid artery plaque (p = 0.025) were more prevalent in DKD group. The two groups also had different antidiabetic prescription patterns (p < 0.001).

A comparison of clinical characteristics of the training and validation cohorts displayed in **Table 1** suggests good inter-cohort comparability. In the included indices, only antidiabetic prescription and the presence of diabetic foot had a p < 0.05. No statistical significance was shown among the three groups in serum albumin level, hemoglobin, urine acid, and systolic blood pressure.

**Table 1:** Clinical characteristics of the training, internal validation, and external cohorts.

Variables	Training cohort (n = 192)	Internal validation (n = 64)	External validation (n = 64)	P
Age, years	61.00 (55.00,69.00)	63.50 (55.00,70.25)	62.00 (53.75,70.25)	0.462b
Diabetic kidney disease				0.226 c
No	70 (36.46)	20 (31.25)	16 (25.00)	
Yes	122 (63.54)	44 (68.75)	48 (75.00)	
Gender				0.820 c
Male	116 (60.42)	37 (57.81)	36 (56.25)	
Female	76 (39.58)	27 (42.19)	28 (43.75)	
Smoking	81 (42.19)	26 (40.62)	26 (40.62)	0.962 c
Diabetic duration, years	14.00 (8.25,20.00)	15.00 (10.00,20.50)	15.50 (10.00,20.00)	0.314 b
Hypertension	139 (72.40)	51 (79.69)	42 (65.62)	0.204 c
HTN grade				0.487 c
None	54 (28.12)	13 (20.31)	21 (32.81)	
I	11 (5.73)	4 (6.25)	3 (4.69)	
II	36 (18.75)	13 (20.31)	6 (9.38)	
III	91 (47.40)	34 (53.12)	34 (53.12)	
Diabetic retinopathy	149 (78.84)	51 (80.95)	52 (81.25)	0.885 c
Peripheral neuropathy	166 (88.77)	61 (96.83)	55 (88.71)	0.152 c
Peripheral vascular disease	134 (72.43)	50 (79.37)	46 (74.19)	0.554 c
Diabetic foot	24 (12.83)	17 (26.98)	12 (19.35)	0.030 c
Coronary heart disease	60 (31.25)	22 (34.38)	23 (35.94)	0.753 c
Cerebrovascular disease	47 (24.48)	22 (34.38)	14 (21.88)	0.209 c
Cerebral infarction	32 (19.05)	11 (18.97)	10 (16.95)	0.935 c
Antidiabetic prescription				0.033 c
Diet control	14 (7.29)	2 (3.12)	0 (0.00)	
Insulin	22 (11.46)	8 (12.50)	14 (21.88)	
OHA	75 (39.06)	20 (31.25)	17 (26.56)	
Both	81 (42.19)	34 (53.12)	33 (51.56)	
Systolic pressure, mmHg	147.00 (131.00,162.00)	148.50 (124.50,161.75)	148.00 (130.50,162.75)	0.960 b
Diastolic pressure, mmHg	79.41 ± 12.86	79.30 ± 14.16	78.69 ± 10.34	0.924 a
Weight, kg	71.97 ± 12.76	75.12 ± 13.94	74.75 ± 11.34	0.133 a
Height, m	1.68 (1.60,1.73)	1.65 (1.60,1.74)	1.67 (1.60,1.72)	0.994 b
BMI, kg/m2	25.30 (23.61,27.89)	26.20 (24.65,29.52)	26.08 (24.22,29.06)	0.065 b
Waist circumference, cm	97.00 (90.00,103.00)	96.00 (90.75,103.50)	97.00 (90.00,102.00)	0.984 b
Waist-to-Hip Ratio	0.96 (0.91,1.00)	0.95 (0.91,1.02)	0.95 (0.90,1.00)	0.841 b
Blood glucose, mmol/L	11.31 ± 3.59	12.60 ± 4.28	11.20 ± 3.30	0.051 a
Total cholesterol, mmol/L	4.65 ± 1.52	4.22 ± 1.53	4.50 ± 1.25	0.126 a
Serum creatinine, μmol/L	77.00 (64.00,132.00)	81.00 (64.50,122.50)	87.00 (60.75,168.00)	0.668 b
Urine Acid, μmol/L	345.00 ± 109.23	336.37 ± 109.99	376.00 ± 143.68	0.148 a
eGFR, mL/min/1.73m2	81.97 (44.94,103.16)	81.45 (49.80,96.78)	69.59 (31.09,96.66)	0.335 b
Cystatin C, mg/L	1.17 (0.92,1.93)	1.27 (0.93,1.98)	1.20 (0.95,2.78)	0.666 b
Urinary albumin, mg	59.26 (10.59,793.67)	106.64 (14.19,902.52)	75.81 (23.71,705.62)	0.542 b
UACR, mg/g	95.00 (20.00,1612.50)	190.00 (32.50,1787.50)	120.00 (40.00,1375.00)	0.684 b
GHbA1c, %	7.90 (6.93,9.40)	8.90 (7.30,10.22)	7.80 (6.90,9.40)	0.127 b
Triglyceride, mmol/L	1.57 (1.08,2.19)	1.47 (1.03,2.00)	1.51 (1.08,2.01)	0.551 b
LDL-C, mmol/L	2.57 ± 0.96	2.39 ± 1.17	2.59 ± 0.90	0.423 a
HDL-C, mmol/L	1.10 (0.89,1.27)	1.02 (0.93,1.25)	1.04 (0.85,1.21)	0.409 b
Albumin, g/L	40.75 (35.35,44.10)	40.30 (38.10,42.85)	40.50 (36.85,43.75)	0.877 b
Haemoglobin, g/L	122.59 ± 27.21	125.38 ± 19.31	121.41 ± 26.26	0.657 a
WBC count, 109/L	6.23 (5.27,7.66)	6.57 (5.81,7.95)	6.43 (5.03,7.90)	0.356 b
Neutrophil count, 109/L	3.90 (3.19,5.30)	4.18 (3.51,5.20)	4.16 (3.05,5.64)	0.588 b
Monocyte count, 109/L	0.39 (0.31,0.49)	0.40 (0.30,0.49)	0.40 (0.32,0.46)	0.955 b
Lymphocyte count	1.67 ± 0.54	1.77 ± 0.68	1.62 ± 0.56	0.348 a
Platelet count, 109/L	224.00 (185.00,276.00)	217.00 (188.50,257.50)	221.00 (184.50,282.00)	0.801 b
NLR	2.52 (1.90,3.23)	2.50 (1.80,3.59)	2.50 (1.88,4.05)	0.778 b
PLR	141.24 (103.40,182.00)	135.52 (99.75,178.60)	144.67 (104.04,204.81)	0.602 b
SII	553.00 (391.53,819.20)	525.59 (373.91,722.44)	546.39 (366.28,1043.33)	0.752 b

BMI, body mass index; HTN, hypertension; OHA, oral hypoglycaemic agent; eGFR, estimated glomerular filtration rate; UACR, urine albumin-to-creatinine ratio; GHBA1c, glycosylated haemoglobin; LDL-C, low-density lipoprotein-cholesterol; HDL-C, high-density lipoprotein-cholesterol; WBC, white blood cell; NLR, neutrophil to lymphocyte ratio; PLR, platelet to lymphocyte ratio; SII, systemic immune inflammation index.

a: ANOVA; b: Kruskal-waills test; c: Chi-square test.

### 3.2 Radiomics and clinical feature selection

**Figure 3:**
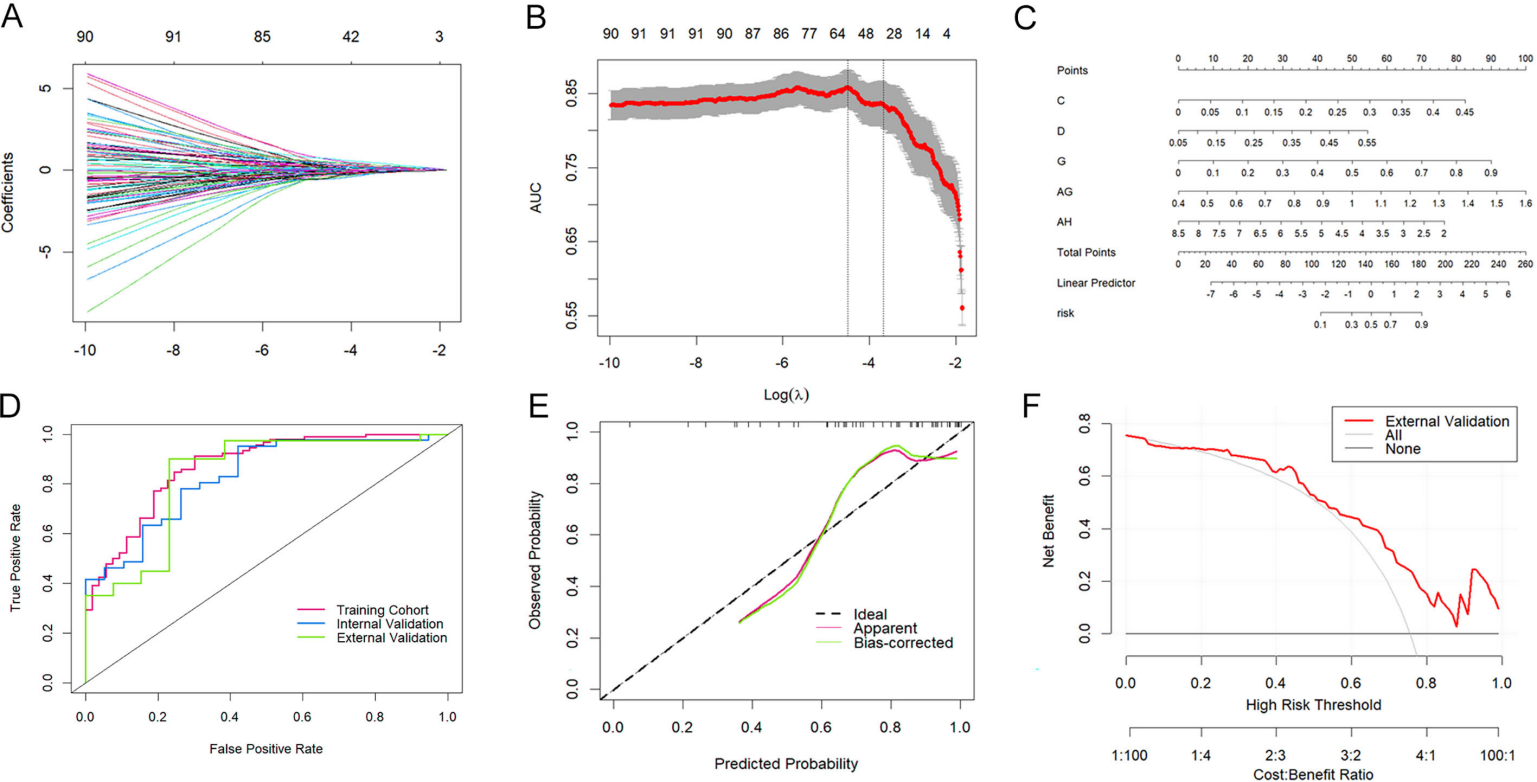
Performance of the radiomics model. **(A)** Coefficient paths across log(λ) values in the LASSO regression. **(B)** 10-fold cross-validation of the LASSO regression. **(C)** Nomogram for selected radiomics features of DKD vs. no-DKD in patients with T2DM. C, RSATArea-auto:original_shape_Elongation; D, RSATArea-auto:wavelet-HHL_glszm_SmallAreaLowGrayLevelEmphasis; G, RenalArea-auto:lbp-3D-k_glszm_SmallAreaEmphasis; AG, PRATVolume-auto:wavelet-HHH_glszm_SmallAreaHighGrayLevelEmphasis; AH, PRATVolume-auto:wavelet-HLL_firstorder_Maximum. **(D)** ROC curves of the radiomics model in the training, internal and external validation cohorts. The red line represents the training cohort curve. The blue line represents the internal validation cohort curve, whereas the green line represents the external validation cohort curve. **(E)** Calibration curves of the nomogram for the external validation cohort. **(F)** Analysis of clinical net benefit of the radiomics model across different decision thresholds to guide interventions in the external validation cohort. LASSO, least absolute shrinkage and selection operator; RSAT, renal sinus adipose tissue; PRAT, perirenal adipose tissue; ROC, receiver operating characteristic; DKD, diabetic kidney disease.

**Figure 4:**
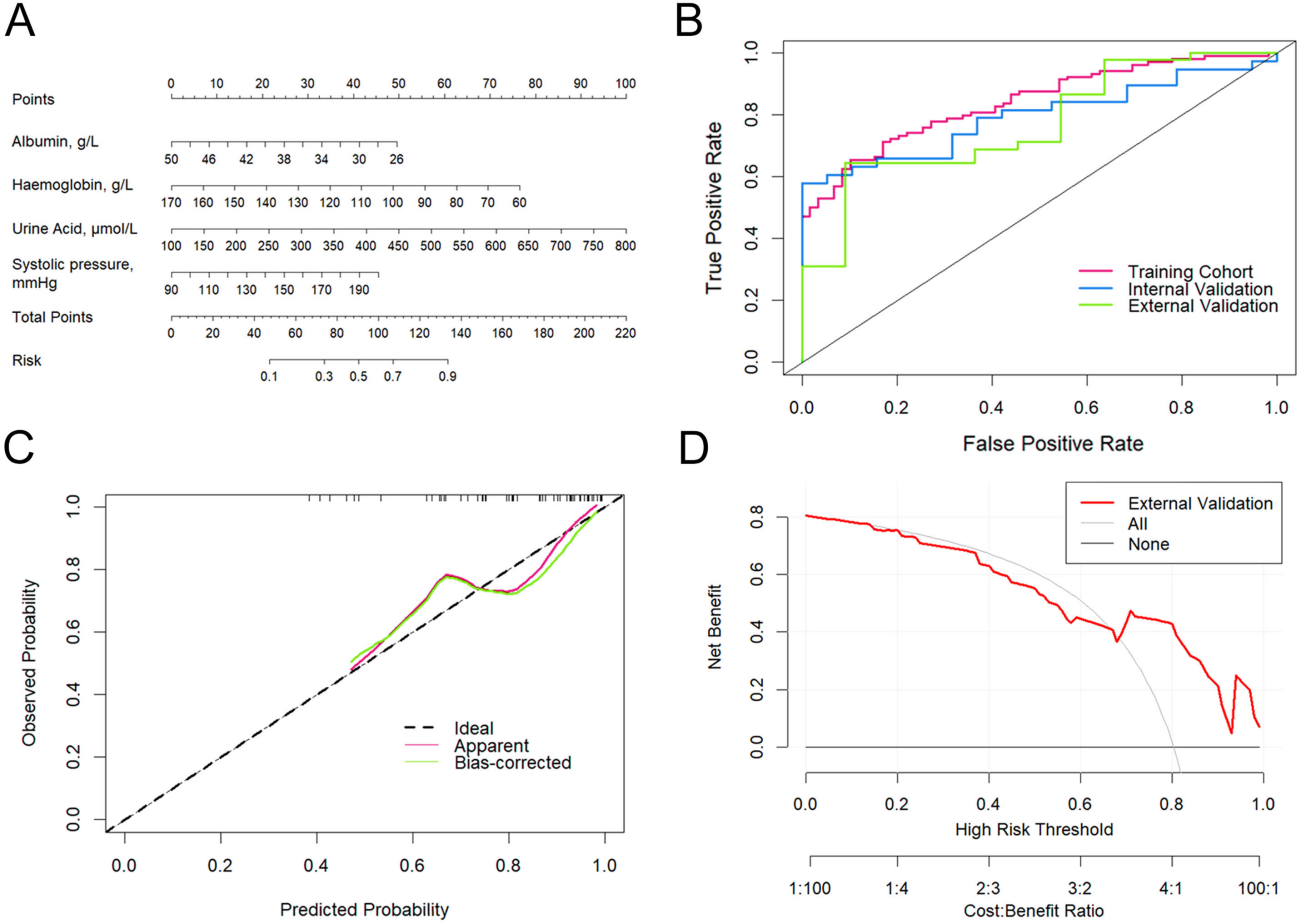
The performance of the clinical model. **(A)** Nomogram for clinical features of DKD vs. no-DKD in patients with T2DM. **(B)** ROC curves of the clinical model in the training, internal, and external validation cohorts. The red line represents the training cohort curve. The blue line represents the internal validation cohort curve, whereas the green line represents the external validation cohort curve. **(C)** Calibration curves of the nomogram for the external validation cohort. **(D)** Analysis of clinical net benefit of the clinical model across different decision thresholds to guide interventions in the external validation cohort. ROC, receiver operating characteristic; T2DM, type 2 diabetes mellitus; DKD, diabetic kidney disease.

**Figure 5:**
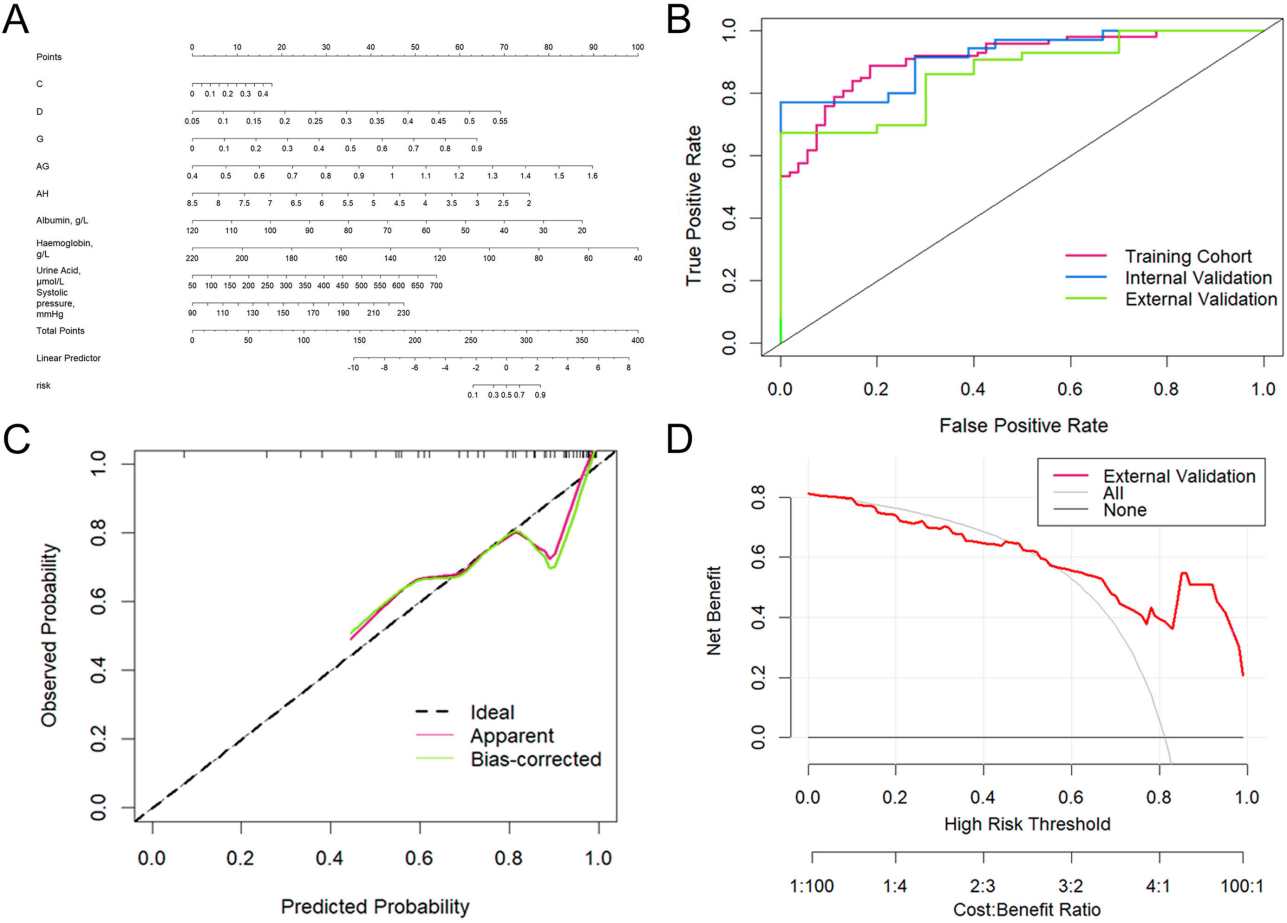
The performance of the combined model. **(A)** Nomogram for integrated variables of DKD vs. no-DKD in patients with T2DM. C, RSATArea-auto:original_shape_Elongation; D, RSATArea-auto:wavelet-HHL_glszm_SmallAreaLowGrayLevelEmphasis; G, RenalArea-auto:lbp-3D-k_glszm_SmallAreaEmphasis; AG, PRATVolume-auto:wavelet-HHH_glszm_SmallAreaHighGrayLevelEmphasis; AH, PRATVolume-auto:wavelet-HLL_firstorder_Maximum. **(B)** ROC curves of the combined model in the training, internal, and external validation cohorts. The red line represents the training cohort curve. The blue line represents the internal validation cohort curve, whereas the green line represents the external validation cohort curve. **(C)** Calibration curves of the nomogram for external validation cohort. **(D)** Analysis of the clinical net benefit of the clinical model across different decision thresholds in the external validation cohort. RSAT, renal sinus adipose tissue; PRAT, perirenal adipose tissue; ROC, receiver operating characteristic; DKD, diabetic kidney disease.

An acceptable inter-observer concordance is suggested as all ICCs were above 0.8. The correlation heatmap for each anatomical compartment is provided in **Supplementary Figure 1**. The LASSO paths and coefficients for the selected features in the compartment-based feature selection are shown in **Supplementary Figures 2**, **3**, respectively. After merging the features selected by each submodel, we employed the LASSO algorithm again to select 34 features from the 117 original features (**Figures 3A, B**). The resultant features consist of 2 features from VAT ROI, 3 from RSAT ROI, 5 from kidney ROI, 2 from PRAT ROI, 3 from SM ROI, 2 from VAT VOI, 4 from SAT VOI, 1 from RSAT VOI, 4 from kidney VOI, 3 from PRAT VOI, and 5 from SM VOI. The coefficients for these features were provided in **Supplementary Table 2**. These variables were further included in univariate and multivariate logistic regression analyses. A total of five features with a p < 0.05 in the multivariate analysis were used as the final modeling variables for the omics model (**Figure 3C**). These features were auto:original_shape_Elongation and auto:wavelet-HHL_glszm_SmallAreaLowGrayLevelEmphasis from RSAT ROI, auto:lbp-3D-k_glszm_SmallAreaEmphasis from kidney ROI, auto:wavelet-HHH_glszm_SmallAreaHighGrayLevelEmphasis and auto:wavelet-HLL_firstorder_Maximum from PRAT VOI. Univariate and Multivariate logistic analysis of clinical variables is shown in **Supplementary Table 4**, according to which albumin, hemoglobin, urine acid, and systolic blood pressure were included for model construction (p < 0.05 in multivariate analysis) (**Figure 4A**). The integrated model was developed based on the variables and features selected above (**Figure 5A**).

### 3.3 Model performance and evaluation

**Figure 6:**
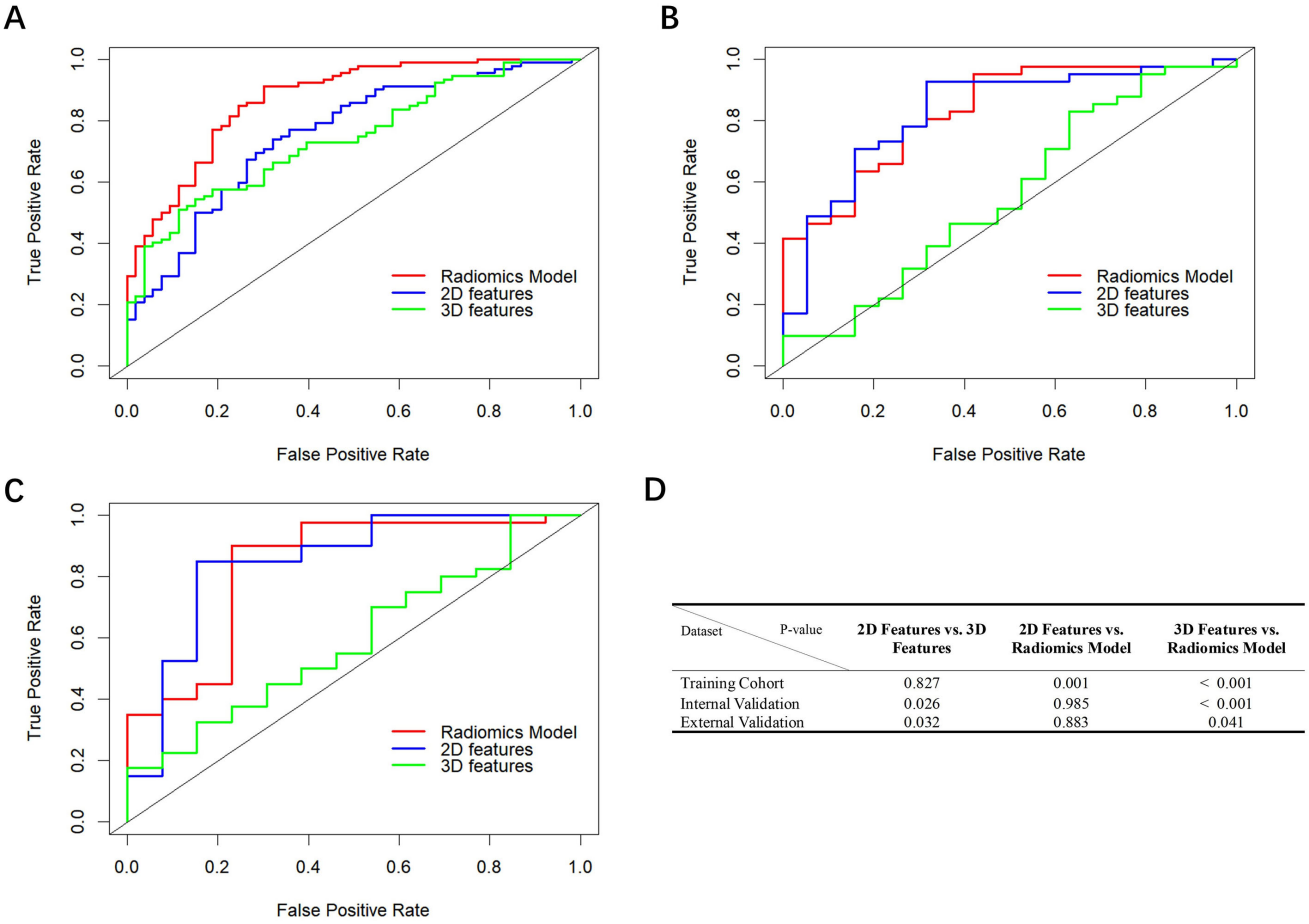
Comparison of features derived from two- and three-dimensional regions on the training **(A),** internal validation **(B)**, and external validation **(C)** cohorts. Comparative analysis by DeLong’s test is displayed in **(D)**. *2D*, two-dimension, *3D*, three-dimension. .

The evaluation of the radiomics, clinical, and integrated model is displayed in **Figures 3**–**5**, respectively. The radiomics model achieved an impressive AUC of 0.869 (95% CI, 0.809–0.929), 0.831 (95% CI, 0.721–0.940), and 0.833 (95% CI, 0.687–0.979) (**Figure 3D**). The ROC curves of the clinical nomogram (training cohort AUC: 0.840, 95% CI: 0.782–0.899; internal validation cohort AUC: 0.794, 95% CI: 0.680–0.908; external validation cohort AUC: 0.770, 95% CI: 0.621–0.919) demonstrate moderate diagnostic performance for the existence of DKD in patients with T2DM (**Figure 4B**). The integrated model achieved high performance, with the highest AUCs in training, internal validation, and external validation being 0.913 (95% CI, 0.868–0.957), 0.919 (95% CI, 0.849–0.989), and 0.867 (95% CI, 0.762–0.973), respectively (**Figure 5B**). As suggested by DeLong’s test, the combined model outperformed the clinical model in all datasets (p < 0.01, p = 0.023, p = 0.021 for training, internal validation, and external validation sets, respectively). No statistical difference was revealed between the radiomics model and the clinical model or the combined model across all datasets (p > 0.05). For all three models, the calibration curve showed good agreement between predictions and observations in the training and validation cohorts (**Figures 3E**, **4C**, **5C**). Calibration curves and DCAs (**Figures 3F**, **4D**, **5D)** of the external validation cohort were provided in each figure. Rest calibration curves and decision curves of each cohort were provided in **Supplementary Figures 4**–**6**. The supplementary figures suggested that the calibration curve of the radiomics model was the most optimistic one, but the combined model possessed a higher net benefit across extended threshold intervals in both groups compared with the radiomics model and the clinical nomogram. To evaluate the individual discriminative ability of each feature, univariate ROC curve analyses were performed in both the training and validation cohorts (**Supplementary Figure 7**). The predictor with the best AUC varies slightly in different datasets, but both kidney- and adipose-derived parameters exhibited moderate discriminative capability. For the radiomics model, two submodels based on ROI and VOI features from the eventual modeling features were created separately and compared (**Figures 6A–D**). The radiomics model surpassed the VOI-derived submodel in AUC in all cohorts, and outstripped ROI-derived submodel in the training cohort. The diagnostic performance of the three models was further compared by calculating sensitivity, specificity, positive predictive value, negative predictive value, and Youden Index, as presented in **Table 2**. For further comparative visualization of these established models, the nomograms and ROCs in the two validation cohorts with marked optimal cutoff points determined by the Youden index are sorted to **Supplementary Figure 8**.

**Table 2:** Diagnostic performance of the three models in training, internal validation, and external cohorts.

Cohort	Model	AUC (95% CI)	SEN	SPE	PPV	NPV	Youden Index
Training cohort	Radiomics	0.869 (0.809, 0.929)	0.913	0.698	0.840	0.822	0.611
	Clinical	0.840 (0.782, 0.899)	0.654	0.898	0.919	0.596	0.552
	Combined	0.913 (0.868, 0.957)	0.889	0.815	0.898	0.800	0.704
Internal validation cohort	Radiomics	0.831 (0.721, 0.940)	0.951	0.579	0.830	0.846	0.530
	Clinical	0.794 (0.680, 0.908)	0.579	1.000	1.000	0.543	0.579
	Combined	0.919 (0.849, 0.989)	0.771	1.000	1.000	0.692	0.771
External validation cohort	Radiomics	0.833 (0.687, 0.979)	0.900	0.769	0.923	0.714	0.669
	Clinical	0.770 (0.621, 0.919)	0.511	0.909	0.958	0.312	0.420
	Combined	0.867 (0.762, 0.973)	0.674	1.000	1.000	0.417	0.674

SEN, sensitivity; SPE, specificity; PPV, positive predictive value; NPV, negative predictive value.

## 4 Discussion

T2DM has reached an epidemic level globally, posing an enormous health and economic burden to its victims along with multiple complications in the prolonged course, the most frequent and severe one being kidney diseases (2, 22). In the United States, DKD affects over 40% of individuals diagnosed with T2DM (23). Intricate pathophysiological mechanisms characterize the initiation and advancement of DKD, but these microscopic differences are barely perceivable from routine medical images. This study aims to establish an imaging-based diagnostic model for DKD and to evaluate the diagnostic value of imaging techniques in patients clinically diagnosed with DKD. The research adopted CT examinations prescribed for patients with T2DM, most frequently for checking cardiovascular or abdominal disease, obviating extra examinations, and requiring only a secondary analysis on routine medical images, avoiding unnecessary medical costs and patient burden.

The current work made innovations in the following aspects. Firstly, this study represents the first effort to integrate radiomics features from multiple anatomical sites—including abdominal muscles, subcutaneous fat, and visceral fat—for the evaluation of DKD. Notably, Liao et al. identified SAT radiodensity as a significant predictor of chronic kidney disease progression; in patients with DKD, the visceral-to-subcutaneous fat ratio and radiodensity of RSAT have also been highlighted as important factors (24). Research involving patients and healthy donors found that deep learning-derived abdominal body composition parameters can reliably estimate creatinine production in both patients and healthy individuals (25). The reviewed literature substantiates our hypothesis that imaging characteristics related to body composition may serve as indicators of renal function. In the LASSO-based feature selection, 16 out of 34 features were derived from SM, SAT, or VAT segmentations. Admittedly, testing the efficacy of an exclusive body-composition radiomics model, incorporating data from traditional imaging measurements, and conducting comparative analyses would provide a more detailed assessment of the utility of radiomics features.

Second, special attention was paid to perirenal radiomics in renal function evaluation. Increased volumes of RSAT and PRAT might result in mechanical compression, which induces decreased tubular flow rate, increased NaCl reabsorption, increased renal blood flow and glomerular filtration rate, and stimulation of renin secretion (26). Animal experiments found that PRAT showed increased pro-inflammatory macrophage infiltration and TNF-α expression and mediates renal artery endothelial dysfunction that predicts renal impairment (27). PRAT may also aggravate renal dysfunction by producing lipotoxicity and hyperactivating the renin-angiotensin-aldosterone system (28). Studies on RSAT biopsies demonstrate an inflammatory and macrophage-enriched profile (29). Although perirenal radiomics has demonstrated value in characterizing urinary tumors, its association with chronic kidney disease remains unexplored in the literature (30, 31).

Most previous studies on chronic kidney diseases have focused solely on renal radiomics (32), which can identify established renal damage but often fail to predict subclinical kidney damage mediated by systemic metabolic disorders. Additionally, some studies have examined skeletal muscle or adipose tissue separately, without combining these analyses with renal radiomics, thereby limiting the ability to distinguish disease states. Tang et al. found that reduced renal sinus fat density is significantly associated with early DKD in patients with T2DM, demonstrating potential utility as a biomarker for risk stratification (AUC = 0.81 for the combined clinical-body composition model) (33). A Japanese prospective cohort study found that renal sinus fat volume index predicted both proteinuria and GFR decline (AUC = 0.769), independent of classical adiposity indices such as BMI or waist circumference (34). This study builds upon CT radiomics of the kidney and perirenal fat. In the feature selection process, it incorporates features of skeletal muscle, subcutaneous, and visceral fat, establishing a comprehensive assessment system that integrates multiple heterogeneous metabolic tissues. Liao et al. adopted clinical factors and SAT radiodensity to predict progression of renal function loss (24). We assume that the kidney and perirenal fat reflect local lipotoxic microenvironments and structural renal damage, while skeletal muscle and different fat depots provide additional information on holistic metabolic risk. Although the final model excluded radiomics features from SM, SAT or VAT, application of this method to predict DKD progression might utilize these features.

Another innovation lies in the methodology for CT radiomics. The association between renal radiomics and chronic kidney disease has been extensively documented (8, 9). CT imaging offers distinct advantages for three-dimensional reconstruction owing to its high spatial resolution. Prior research has extracted morphological features from ROIs within three-dimensional kidney models, revealing their diagnostic relevance in assessing glomerular filtration rate decline (35). In the present study, ROIs were delineated in both two-dimensional and three-dimensional formats, enabling the acquisition of more comprehensive information from the same tissue, and the ultimate modeling features encompass those retrieved from both two-dimensional and three-dimensional regions, and the combined model exhibited statistically better performance than the three-dimensional submodel. Restricted by the sample size and limited number of features, the superiority of this strategy awaits additional validation in expanded datasets. In this study, clinical, radiomics, and combined models were constructed to identify DKD in patients with T2DM. UACR and eGFR are indicators of kidney function, whereas radiomics features represent structural phenotypes. The results suggest that 2 radiomics features from RSAT, 1 feature from the kidneys, and 2 features from PRAT are related to DKD diagnosis, indicating that alterations in eGFR and UACR might accompany macroscopic or microscopic changes in the tissues above, revealing a structural-functional correspondence. As a pioneering effort to integrate a renal and body-composition radiomics model, our study reveals an intrinsic association among metabolic disorders, body-composition changes, and renal impairment. Future research could use biopsy as the gold standard to investigate whether these omics-based indicators hold diagnostic value in atypical DKD patients with abnormal renal biopsy findings but unclear UACR or eGFR alterations. Besides, since our model achieves high diagnostic accuracy in the typical DKD population defined by eGFR and UACR, it retains significant value for routine clinical practice by serving as a non-invasive auxiliary screening tool for opportunistic DKD risk assessment in T2DM patients undergoing abdominal CT scans. However, admittedly, adoption of UACR and eGFR as the surrogate diagnostic standard for DKD instead of renal biopsy histopathology might cause diagnostic bias, for instance, confounded eGFR values induced by muscle mass and medications, or false albuminuria, which may lead to misclassification of participants and compromise the reliability of the predictive model.

A lack of pathophysiological interpretation is a natural drawback of radiomics. The features selected for model development in this study are amenable to pathological explanations grounded in observations from previous studies. Lee et al. demonstrated that the anteroposterior diameter of the renal sinus, which reflects morphological distortion of the renal sinus, expanded in chronic kidney disease (36). The present model used the RSAT original_shape_Elongation feature, representing the relationship between the two largest principal components of the ROI shape (37), to detect DKD, which is consistent with the radiological findings described in the literature. Due to the intricate pathological development, DKD exhibits multifaceted microscopic alterations, including hypertrophy of glomeruli, thickening of the glomerular basement membrane, and expansion of the mesangial matrix (38). A previous study has correlated CT radiomic features with kidney biopsy characteristics (39). We thus assume that some features are associated with microscopic alterations in the ROIs. For example, SmallAreaLow/HighGrayLevelEmphasis features detect small, low/high-gray-level regions with unique features. Changes in these features might reflect pixel intensities and thereby, heterogeneity and localized inflammation in the ROI. In summary, the CT radiomic parameters used in this study align with conclusions in the literature and the pathological changes during the progression of DKD, demonstrating interpretability and rationality.

A limitation of the present study is its retrospective design, which precluded investigation of the association between radiomics and renal biopsy pathology. Since the study population was selected through opportunistic use of CT scans rather than predefined DKD staging, the findings—while aligning with anticipated outcomes—may overstate the efficacy of early DKD detection. Contrast-enhanced CT information reflecting renal microperfusion was not investigated, prohibiting evaluation of microcirculatory damage in DKD patients. Also, a 5 mm slice thickness compromises the longitudinal resolution of three-dimensional VOIs and distorts volumetric texture metrics, exerting negative effects on the extraction of two- and three-dimensional radiomic features. The association of these radiomics features with the DKD severity stages by subgroup analysis or progression of kidney impairment warrants further exploration. The development of artificial intelligence holds promise to automate segmentation and feature extraction at scale, simplifying workflows and facilitating clinical translation. Admittedly, an external validation cohort of merely 64 participants would lead to a wide 95% CI of AUC and restrict the generalizability of our models. Moreover, an independent prospective cohort for external validation might strengthen direct clinical translation of the established model.

In summary, the present research innovatively established a clinical-omics-integrated diagnostic model based on body composition, renal and perirenal radiological features to identify DKD in T2DM patients, demonstrating that the discussed radiomics features are valuable for DKD diagnosis. This integrated model realizes opportunistic identification for DKD patients via routine CT. For T2DM patients who accept opportunistic abdominal CT for various reasons, a positive radiomics finding might suggest kidney function examinations. Although laboratory tests are commonly used for DKD screening, this model has the potential to quickly recommend specialized urological care, particularly for T2DM patients visiting other medical departments and lacking specialized endocrinological examinations, and subsequently, facilitate earlier, personalized therapeutic strategies to preserve renal function and ameliorate long-term outcomes.

## Data Availability

The raw data supporting the conclusions of this article will be made available by the authors, without undue reservation.
