# Supplementary material for: CT-based renal and body-composition radiomics model to improve the detection ability of diabetic kidney disease in patients with type 2 diabetes mellitus
Source: Front Endocrinol (Lausanne). 2026 Aug 3;17:1904758. doi: 10.3389/fendo.2026.1904758 (PMC13477953; doi:10.3389/fendo.2026.1904758)
Supplement: Supplementary file 1 [file Supplementaryfile1.docx]

**SUPPLEMENTARY MATERIALS**

**Part I Supplementary Tables**

Supplementary Table 1 Cut-off values of anatomical region segmentation

Supplementary Table 2 Clinical characteristics of the enrolled patients

Supplementary Table 3 Coefficients of the fused LASSO model

Supplementary Table 4 Comparisons of radiomic feature distributions between DKD and non-DKD subgroups

Supplementary Table 5 Results of Logistic regression analysis for clinical variables

**Part II Supplementary Figures**

Supplementary Figure 1 Heatmap of radiomics features from regions or volumes of interest

Supplementary Figure 2 LASSO paths of the model-based feature selection process

Supplementary Figure 3 Coefficients in each LASSO model

Supplementary Figure 4 The evaluation of radiomics model in training and internal validation cohorts

Supplementary Figure 5 The evaluation of clinical model in training and internal validation cohorts

Supplementary Figure 6 The evaluation of combined model in training and internal validation cohorts

Supplementary Figure 7 Comparison of univariate features by area under the receiver operating characteristic curve

Supplementary Figure 8 Comparison of nomogram and model performance on the validation cohorts across the three models

**Part I Supplementary Tables**

**Supplementary Table 1** Cut-off values of anatomical region segmentation

| Region | Cut-off value (HU) |
| --- | --- |
|  |  |
| SM | -30—150 |
| SAT | -190—-30 |
| VAT | -150—-50 |
| PRAT | -150—-50 |
| RSAT | -150—-50 |
| Kidneys | -30—150 |

**Note:** SM, skeletal muscle; SAT, subcutaneous adipose tissue; VAT, visceral adipose tissue; PRAT perirenal adipose tissue; RSAT, renal sinus adipose tissue.

**Supplementary Table 2** Clinical characteristics of the enrolled patients

| Variables | Total  (n = 320) | No DKD  (n = 106) | DKD  (n = 214) | *P* |
| --- | --- | --- | --- | --- |
|  |  |  |  |  |
| Age, years | 61.67 ± 11.09 | 61.70 ± 9.74 | 61.65 ± 11.73 | 0.972 ^a^ |
| Gender |  |  |  | 0.563 ^b^ |
| Male | 189 (59.06) | 65 (61.32) | 124 (57.94) |  |
| Female | 131 (40.94) | 41 (38.68) | 90 (42.06) |  |
| BMI, kg/m^2^ | 25.56 (23.83, 28.41) | 25.40 (23.63, 27.50) | 25.71 (24.09, 29.30) | 0.173 ^c^ |
| Waist-to-hip ratio | 0.96 (0.91, 1.00) | 0.95 (0.91, 0.99) | 0.96 (0.90, 1.00) | 0.376 ^c^ |
| Diabetic duration, years | 14.50 (9.00, 20.00) | 12.00 (7.25, 20.00) | 15.00 (10.00, 20.00) | 0.007 ^c^ |
| Smoking | 133 (41.56) | 39 (36.79) | 94 (43.93) | 0.223 ^b^ |
| Hypertension | 232 (72.50) | 62 (58.49) | 170 (79.44) | <.001 ^b^ |
| HTN grade |  |  |  | <.001 ^b^ |
| None | 88 (27.50) | 44 (41.51) | 44 (20.56) |  |
| I | 18 (5.62) | 5 (4.72) | 13 (6.07) |  |
| II | 55 (17.19) | 18 (16.98) | 37 (17.29) |  |
| III | 159 (49.69) | 39 (36.79) | 120 (56.07) |  |
| Diabetic retinopathy | 252 (79.75) | 84 (79.25) | 168 (80.00) | 0.875 ^b^ |
| Peripheral neuropathy | 282 (90.38) | 99 (93.40) | 183 (88.83) | 0.196 ^b^ |
| Peripheral vascular disease | 230 (74.19) | 77 (72.64) | 153 (75.00) | 0.653 ^b^ |
| Diabetic foot | 53 (16.99) | 13 (12.26) | 40 (19.42) | 0.111 ^b^ |
| Coronary heart disease | 105 (32.81) | 39 (36.79) | 66 (30.84) | 0.286 ^b^ |
| Cerebrovascular disease | 83 (25.94) | 30 (28.30) | 53 (24.77) | 0.497 ^b^ |
| Cerebral infarction | 53 (18.60) | 10 (13.51) | 43 (20.38) | 0.192 ^b^ |
| Antidiabetic prescription |  |  |  | <.001 ^b^ |
| Diet control | 16 (5.00) | 6 (5.66) | 10 (4.67) |  |
| Insulin | 44 (13.75) | 5 (4.72) | 39 (18.22) |  |
| OHA | 112 (35.00) | 52 (49.06) | 60 (28.04) |  |
| Both | 148 (46.25) | 43 (40.57) | 105 (49.07) |  |
| Systolic pressure, mmHg | 146.74 ± 23.21 | 138.27 ± 18.73 | 150.93 ± 24.09 | <.001 ^b^ |
| Diastolic pressure, mmHg | 79.24 ± 12.64 | 78.27 ± 10.89 | 79.72 ± 13.43 | 0.301 ^a^ |
| Waist circumference, cm | 97.04 ± 10.11 | 97.29 ± 8.73 | 96.88 ± 10.95 | 0.744 ^a^ |
| LDL-C, mmol/L | 2.54 ± 1.00 | 2.49 ± 0.89 | 2.56 ± 1.05 | 0.601 ^a^ |
| HDL-C, mmol/L | 1.09 ± 0.31 | 1.08 ± 0.25 | 1.10 ± 0.34 | 0.627 ^a^ |
| Haemoglobin, g/L | 122.91 ± 25.57 | 137.46 ± 18.08 | 115.68 ± 25.70 | <.001 ^a^ |
| Urine acid, μmol/L | 349.41 ± 117.35 | 303.01 ± 94.04 | 371.52 ± 121.06 | <.001 ^a^ |
| Serum creatinine, μmol/L | 79.00 (63.00, 131.25) | 65.00 (57.00, 76.00) | 105.00 (70.50, 213.00) | <.001 ^c^ |
| eGFR, mL/min/1.73m^2^ | 80.00 (43.15, 100.52) | 97.15 (87.84, 107.64) | 59.14 (30.05, 86.59) | <.001 ^c^ |
| Cystatin C, mg/L | 1.19 (0.93, 2.02) | 0.93 (0.82, 1.05) | 1.54 (1.10, 2.57) | <.001 ^c^ |
| Urinary albumin, mg | 70.00 (13.16, 842.93) | 9.78 (6.56, 20.71) | 280.14 (69.62, 1629.82) | <.001 ^c^ |
| UACR, mg/g | 100.00 (30.00, 1600.00) | 20.00 (10.00, 30.00) | 580.00 (100.00, 3380.00) | <.001 ^c^ |
| Blood glucose, mmol/L | 10.84 (8.77, 13.78) | 12.07 (9.48, 13.98) | 10.54 (8.60, 13.59) | 0.049 ^c^ |
| GHbA1c, % | 8.00 (6.90, 9.50) | 8.40 (7.25, 9.60) | 7.90 (6.80, 9.40) | 0.169 ^c^ |
| Total cholesterol, mmol/L | 4.37 (3.46, 5.33) | 4.27 (3.59, 5.06) | 4.42 (3.45, 5.47) | 0.341 ^c^ |
| Triglyceride, mmol/L | 1.52 (1.05, 2.13) | 1.32 (1.01, 2.01) | 1.60 (1.11, 2.17) | 0.116 ^c^ |
| Albumin, g/L | 40.50 (35.90, 43.80) | 42.90 (40.45, 45.45) | 39.20 (33.55, 42.95) | <.001 ^c^ |
| WBC count, 10^9^/L | 6.42 (5.29, 7.78) | 6.22 (5.13, 7.36) | 6.52 (5.30, 7.83) | 0.249 ^c^ |
| Neutrophil count, 10^9^/L | 4.06 (3.20, 5.37) | 3.89 (3.09, 4.83) | 4.16 (3.23, 5.54) | 0.076 ^c^ |
| Monocyte count, 10^9^/L | 0.39 (0.31, 0.49) | 0.38 (0.31, 0.44) | 0.40 (0.31, 0.51) | 0.176 ^c^ |
| Platelet count, 10^9^/L | 221.00 (185.00, 273.00) | 224.00 (189.00, 256.00) | 219.00 (182.00, 284.00) | 0.579 ^c^ |
| NLR | 2.51 (1.87, 3.48) | 2.10 (1.66, 3.08) | 2.70 (1.95, 3.68) | <.001 ^c^ |
| PLR | 141.31 (103.27, 179.96) | 124.07 (100.89, 162.80) | 146.74 (107.18, 198.23) | 0.004 ^c^ |
| SII | 545.20 (380.51, 882.21) | 494.00 (351.49, 695.86) | 603.10 (400.25, 914.33) | 0.003 ^c^ |

**Note:** BMI, body mass index; HTN, hypertension; OHA, oral hypoglycaemic agent; eGFR, estimated glomerular filtration rate; UACR, urine albumin-to-creatinine ratio; GHBA1c, glycosylated haemoglobin; LDL-C, low-density lipoprotein-cholesterol; HDL-C, high-density lipoprotein-cholesterol; WBC, white blood cell; NLR, neutrophil to lymphocyte ratio; PLR, platelet to lymphocyte ratio; SII, systemic immune inflammation index

a: t-test, b: Chi-square test, c: Mann-Whitney test.

**Supplementary Table 3** Coefficients of the fused LASSO model

| ROI/VOI | Variable | Coefficient |
| --- | --- | --- |
| VATArea | auto__exponential_glrlm_RunLengthNonUniformity | 0.010866 |
| VATArea | auto__lbp-3D-k_glszm_ZonePercentage | 0.29054 |
| RSATArea | auto__original_shape_Elongation | 0.147476 |
| RSATArea | auto__wavelet-HHL_glszm_SmallAreaLowGrayLevelEmphasis | 0.379209 |
| RSATArea | auto__wavelet-LHH_glszm_SmallAreaHighGrayLevelEmphasis | -0.10669 |
| KidneyArea | auto__lbp-2D_firstorder_Median | -0.2829 |
| KidneyArea | auto__lbp-3D-k_glszm_SmallAreaEmphasis | 0.501472 |
| KidneyArea | auto__wavelet-HHH_glszm_ZoneEntropy | -0.15948 |
| KidneyArea | auto__wavelet-HHH_ngtdm_Coarseness | 0.19021 |
| KidneyArea | auto__wavelet-LLH_gldm_SmallDependenceHighGrayLevelEmphasis | 0.030665 |
| PRATArea | auto__square_firstorder_Skewness | 0.01709 |
| PRATArea | auto__wavelet-LLL_firstorder_Energy | -0.00977 |
| SMArea | auto__wavelet-HHH_firstorder_Entropy | 0.159589 |
| SMArea | auto__wavelet-HLH_firstorder_Mean | 0.085531 |
| SMArea | auto__wavelet-LHL_firstorder_Skewness | -0.18592 |
| SMVolume | auto__wavelet-HHH_firstorder_Skewness | 0.101231 |
| SMVolume | auto__wavelet-HHH_glszm_HighGrayLevelZoneEmphasis | 0.249404 |
| SMVolume | auto__wavelet-LHH_glcm_SumEntropy | -0.22083 |
| SMVolume | auto__wavelet-LHL_glcm_DifferenceEntropy | -0.1246 |
| SMVolume | auto__wavelet-LLH_glszm_GrayLevelNonUniformityNormalized | -0.0363 |
| VATVolume | auto__lbp-3D-k_glszm_SizeZoneNonUniformityNormalized | -0.00827 |
| VATVolume | auto__wavelet-HLL_glcm_SumEntropy | 0.24493 |
| SATVolume | auto__wavelet-HLH_glszm_GrayLevelNonUniformityNormalized | 0.082696 |
| SATVolume | auto__wavelet-HHH_glrlm_HighGrayLevelRunEmphasis | 0.219751 |
| SATVolume | auto__wavelet-LHL_glrlm_HighGrayLevelRunEmphasis | -0.01499 |
| SATVolume | auto__wavelet-LHH_glcm_SumSquares | 0.096795 |
| RSATVolume | auto__lbp-3D-k_glcm_Correlation | -0.0231 |
| KidneyVolume | auto__lbp-2D_firstorder_Median | -0.14973 |
| KidneyVolume | auto__wavelet-LLH_firstorder_Skewness | 0.175878 |
| KidneyVolume | auto__wavelet-LLH_glrlm_LongRunHighGrayLevelEmphasis | -0.12 |
| KidneyVolume | auto__wavelet-LLH_glszm_GrayLevelNonUniformityNormalized | -0.20936 |
| PRATVolume | auto__wavelet-HHH_glszm_SmallAreaHighGrayLevelEmphasis | 0.346127 |
| PRATVolume | auto__wavelet-HLL_firstorder_Maximum | -0.24245 |
| PRATVolume | auto__wavelet-LLH_glszm_GrayLevelNonUniformityNormalized | -0.10183 |

**Note:** VATArea, visceral adipose tissue area; RSATArea, renal sinus adipose tissue area; PRATArea, perirenal adipose tissue area; SMArea, skeletal muscle area; SMVolume, skeletal muscle volume; VATVolume, visceral adipose tissue volume; SATVolume, subcutaneous adipose tissue volume; RSATVolume, renal sinus adipose tissue volume; PRATVolume, perirenal adipose tissue volume.

**Supplementary Table 4** Comparisons of radiomic feature distributions between DKD and non-DKD subgroups

| Variables | Total  (n = 320) | No DKD  (n = 106) | DKD  (n = 214) | *P* |
| --- | --- | --- | --- | --- |
|  |  |  |  |  |
| RSATArea-auto__original_shape_Elongation | 0.09 (0.08, 0.11) | 0.09 (0.07, 0.10) | 0.09 (0.08, 0.11) | 0.003 |
| RSATArea-auto__wavelet-HHL_glszm_SmallAreaLowGrayLevelEmphasis | 0.28 (0.23, 0.33) | 0.25 (0.21, 0.32) | 0.29 (0.24, 0.34) | <.001 |
| RenalArea-auto__lbp-3D-k_glszm_SmallAreaEmphasis | 0.52 (0.45, 0.57) | 0.47 (0.33, 0.54) | 0.53 (0.50, 0.59) | <.001 |
| PRATVolume-auto__wavelet-HHH_glszm_SmallAreaHighGrayLevelEmphasis | 0.72 (0.65, 0.80) | 0.69 (0.62, 0.76) | 0.73 (0.66, 0.82) | <.001 |
| PRATVolume-auto__wavelet-HLL_firstorder_Maximum | 5.33 (4.62, 6.10) | 5.45 (4.98, 6.19) | 5.16 (4.45, 6.08) | 0.04 |

**Note:** RSATArea, renal sinus adipose tissue area; PRATVolume, perirenal adipose tissue volume.

**Supplementary Table 5** Results of Logistic regression analysis for clinical variables

|  | Univariate analysis | | | Multivariate analysis | | |
| --- | --- | --- | --- | --- | --- | --- |
| Variables | OR | 95%CI | *P* | OR | 95%CI | *P* |
| Gender | 0.87 | 0.54, 1.40 | 0.563 |  |  |  |
| Age, years | 1.00 | 0.98, 1.02 | 0.973 |  |  |  |
| Antidiabetic prescription, n (%) |  |  |  |  |  |  |
| Insulin | 4.68 | 1.19, 19.51 | 0.028 | 1.90 | 0.23, 14.68 | 0.536 |
| OHA | 0.69 | 0.22, 1.99 | 0.504 | 0.54 | 0.0938, 2.62 | 0.456 |
| Both insulin and OHA | 1.47 | 0.47, 4.20 | 0.485 | 1.36 | 0.23, 6.88 | 0.719 |
| Smoking, n (%) | 1.35 | 0.84, 2.18 | 0.224 |  |  |  |
| Hypertension, n (%) | 2.74 | 1.65, 4.58 | <0.001 | 1.22 | 0.60, 2.45 | 0.586 |
| Systolic pressure, mmHg | 1.03 | 1.01, 1.04 | <0.001 | 1.03 | 1.01, 1.04 | 0.001 |
| Diastolic pressure, mmHg | 1.01 | 0.99, 1.03 | 0.334 |  |  |  |
| Height, m | 0.86 | 0.06, 11.81 | 0.910 |  |  |  |
| Weight, kg | 1.01 | 0.99, 1.03 | 0.194 |  |  |  |
| BMI | 1.05 | 0.99, 1.12 | 0.103 |  |  |  |
| Waist circumference, cm | 1.00 | 0.97, 1.02 | 0.754 |  |  |  |
| Waist-to-hip ratio | 3.32 | 0.87, 194.66 | 0.554 |  |  |  |
| Diabetes duration, years | 1.04 | 1.01, 1.08 | 0.005 | 1.01 | 0.96, 1.05 | 0.782 |
| Diabetic retinopathy, n (%) | 1.05 | 0.58, 1.85 | 0.875 |  |  |  |
| Peripheral neuropathy, n (%) | 0.56 | 0.22, 1.30 | 0.200 |  |  |  |
| Peripheral vascular disease, n (%) | 1.13 | 0.66, 1.91 | 0.653 |  |  |  |
| Diabetic foot, n (%) | 1.72 | 0.90, 3.50 | 0.114 |  |  |  |
| Coronary heart disease, n (%) | 0.77 | 0.47, 1.25 | 0.286 |  |  |  |
| Cerebrovascular disease, n (%) | 0.83 | 0.50, 1.42 | 0.497 | 0.82 | 0.39, 1.70 | 0.584 |
| Cerebral infarction, n (%) | 1.64 | 0.80, 3.63 | 0.195 |  |  |  |
| Blood glucose, mmol/L | 0.94 | 0.88, 1.00 | 0.050 |  |  |  |
| GHbA1c, % | 0.92 | 0.81, 1.04 | 0.162 |  |  |  |
| Total cholesterol, mmol/L | 1.13 | 0.96, 1.34 | 0.155 |  |  |  |
| Triglyceride, mmol/L | 1.05 | 0.91, 1.24 | 0.503 |  |  |  |
| LDL-C, mmol/L | 1.07 | 0.84, 1.36 | 0.600 |  |  |  |
| HDL-C, mmol/L | 1.19 | 0.55, 2.63 | 0.659 |  |  |  |
| Albumin, g/L | 0.88 | 0.84, 0.93 | 0.000 | 1.22 | 0.85, 0.98 | 0.036 |
| Haemoglobin, g/L | 0.96 | 0.94, 0.97 | 0.000 | 0.98 | 0.96, 0.99 | 0.013 |
| Urine Acid, μmol/L | 1.01 | 1.00, 1.01 | 0.000 | 1.01 | 1.00, 1.01 | <0.001 |
| White blood cell count | 1.08 | 0.98, 1.22 | 0.155 |  |  |  |
| Neutrophil count | 1.05 | 0.99, 1.16 | 0.289 |  |  |  |
| Lymphocyte count | 0.52 | 0.34, 0.79 | 0.002 | 0.67 | 0.27, 1.59 | 0.370 |
| Monocyte count | 3.82 | 1.12, 18.15 | 0.071 |  |  |  |
| Platelet count | 1.00 | 1.00, 1.00 | 0.240 |  |  |  |
| NLR | 1.20 | 1.05, 1.43 | 0.021 | 0.85 | 0.59, 1.28 | 0.369 |
| PLR | 1.00 | 1.00, 1.01 | 0.009 | 1.00 | 0.99, 1.01 | 0.608 |
| SII | 1.00 | 1.00, 1.00 | 0.033 | 1.00 | 1.00, 1.00 | 0.370 |

**Note:** OHA, oral hypoglycaemic agent; BMI, body mass index; GHBA1c, glycosylated haemoglobin; LDL-C, low-density lipoprotein-cholesterol; HDL-C, high-density lipoprotein-cholesterol; NLR, neutrophil to lymphocyte ratio; PLR, platelet to lymphocyte ratio; SII, systemic immune inflammation index

**Part II Supplementary Figures**

**Supplementary Figure 1** Correlation heatmap visualizing collinearity.

*RSAT*, renal sinus adipose tissue. *PRAT*, perirenal adipose tissue. *VAT*, visceral adipose tissue. *SAT*, subcutaneous adipose tissue. *SM*, skeletal muscle. *ROI*, region of interest. *VOI*, volume of interest.

**Supplementary Figure 2** LASSO paths of the model-based feature selection process.

*RSAT*, renal sinus adipose tissue. *PRAT*, perirenal adipose tissue. *VAT*, visceral adipose tissue. *SAT*, subcutaneous adipose tissue. *SM*, skeletal muscle. *ROI*, region of interest. *VOI*, volume of interest.

**Supplementary Figure 3** Coefficients in each LASSO model.

*RSAT*, renal sinus adipose tissue. *PRAT*, perirenal adipose tissue. *VAT*, visceral adipose tissue. *SAT*, subcutaneous adipose tissue. *SM*, skeletal muscle. *ROI*, region of interest. *VOI*, volume of interest.

**Supplementary Figure 4** The evaluation of radiomics model in training and internal validation cohorts. Calibration curves of training cohort **(A)** and internal validation cohort **(B)**, and decision curve analysis of training cohort **(C)** and internal validation cohort **(D)** are displayed.

**Supplementary Figure 5** The evaluation of clinical model in training **(A)** and internal validation cohort **(B)**, and decision curve analysis of training cohort **(C)** and internal validation cohort **(D)** are displayed.

**Supplementary Figure 6** The evaluation of combined model in training and internal validation cohorts. Calibration curves of training cohort **(A)** and internal validation cohort **(B)**, and decision curve analysis of training cohort **(C)** and internal validation cohort **(D)** are displayed.

**Supplementary Figure 7** Comparison of univariate radiomics features by area under the receiver operating characteristic curve (AUROC) in the training **(A)**, internal validation **(B)**, and external validation **(C)** cohorts. C, RSATArea-auto__original_shape_Elongation; D, RSATArea-auto__wavelet-HHL_glszm_SmallAreaLowGrayLevelEmphasis; G, RenalArea-auto__lbp-3D-k_glszm_SmallAreaEmphasis; AG, PRATVolume-auto__wavelet-HHH_glszm_SmallAreaHighGrayLevelEmphasis; AH, PRATVolume-auto__wavelet-HLL_firstorder_Maximum.

**Supplementary Figure 8** Comparison of nomogram and model performance on the validation cohorts across the three models. **(A)** Clinical nomogram. **(B)** Radiomics nomogram. **(C)** Combined nomogram. The ROCs in the internal validation cohorts with marked optimal cutoff points determined by the Youden index are displayed in **(D)**, **(E)**, and **(F)** for the clinical, radiomics, and the combined model, respectively. Those in the external validation cohorts are shown in **(G)**, **(H)**, and **(I)** for the clinical, radiomics, and the combined model, respectively.
